# Property-governed performance of platinum-modified titania photocatalysts

**DOI:** 10.3389/fchem.2022.972494

**Published:** 2022-09-23

**Authors:** Kunlei Wang, Ewa Kowalska

**Affiliations:** Institute for Catalysis (ICAT), Hokkaido University, Sapporo, Japan

**Keywords:** platinum, titania, photocatalysis, surface modifacation, photodeposition

## Abstract

Titania is probably the most widely investigated semiconductor photocatalyst because of various advantages, such as high activity, thermal and chemical stability, low price, abundance, and negligible toxicity. However, pristine titania is also characterized by charge carriers’ recombination, and thus lower quantum yields of photocatalytic reactions than theoretical 100%. Moreover, its wide bandgap, despite being recommended for excellent redox properties, means also inactivity under visible part of solar radiation. Accordingly, titania has been surface modified, doped and coupled with various elements/compounds. For example, platinum deposited on the surface of titania has shown to improve both UV activity and the performance under vis. Although the studies on titania modification with platinum started almost half a century ago, and huge number of papers have been published up to now, it is unclear which properties are the most crucial and recommended to obtain highly efficient photocatalyst. In the literature, the opposite findings could be found on the property-governed activities that could result from huge differences in the reaction systems, and also examined photocatalysts. Considering the platinum properties, its content, the size of nanoparticles and the oxidation state, must be examined. Obviously, the characteristics of titania also influence the resultant properties of deposited platinum, and thus the overall photocatalytic performance. Although so many reports on Pt/TiO_2_ have been published, it is hardly possible to give indispensable advice on the recommended properties. However, it might be concluded that usually fine platinum NPs uniformly deposited on the titania surface result in high photocatalytic activity, and thus in the low optimal content of necessary platinum. Moreover, the aggregation of titania particles might also help in the lowering the necessary platinum amount (even to 0.2 wt%) due to the interparticle electron transfer mechanism between titania particles in one aggregate. In respect of platinum state, it is thought that it is highly substrate-specific case, and thus either positively charged or zero valent platinum is the most recommended. It might be concluded that despite huge number of papers published on platinum-modified titania, there is still a lack of comprehensive study showing the direct correlation between only one property and the resultant photocatalytic activity.

## Introduction

In 1839, Becquerel reported that a silver chloride (AgCl) electrode in acidic solution exposed to sunlight could be a source of electricity, i.e., the photovoltaic effect − also known as the “Becquerel effect” (Becquerel, 1839). Almost a century later (1921), the first report on “photocatalysis” was published by Renz, which presented the pigment fading, i.e., showing that irradiated oxides could decompose organic dyes and binders of paints (Renz, 1921). Although, these were the first reports on the light-activated reactions, the real interest in this topic has started with the famous paper by Fujishima and Honda on UV-initiated evolution of oxygen and hydrogen (water splitting) on titania and platinum electrodes, respectively (Fujishima and Honda, 1972). Since then, many reports have been published on the activity enhancement, mechanism clarifications (for various reactions), property-governed performance, and possible applications, including several crucial review papers and even books (Fox and Dulay, 1993; Hoffmann et al., 1995; Abe, 2010; Ohtani, 2010; Ibhadon and Fitzpatrick, 2013; Pichat, 2013; Marci et al., 2019; Garcia Lopez et al., 2021; Strunk, 2021).

**FIGURE 1:**
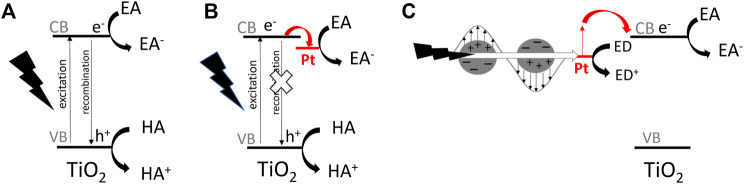
The schematic drawings of simplified mechanisms for photocatalytic reactions on titania-based photocatalyst: **(A)** pristine titania under UV excitation, **(B)** Pt-modified titania under UV excitation, **(C)** Pt-modified titania under vis excitation (considering an electron transfer mechanism); EA—electron acceptor, ED-electron donor, HA—hole acceptor.

Considering the basic mechanism of heterogeneous photocatalysis, the “photocatalyst” (usually oxide semiconductor) is excited under irradiation with light of energy equal or larger than its bandgap, i.e., photo-generated electrons are transferred from the valence band (VB) to the conduction band (CB), as shown in Figure 1A. The electrons and positive holes (simultaneously formed in VB) initiate reactions that cannot proceed spontaneously without photocatalyst (and without irradiation). Moreover, same as in the case of catalytic (“dark”) reactions, photocatalysts (like catalysts) should not be changed during reactions. Although photocatalytic and catalytic reactions are similar, considering the necessary stability of (photo)catalysts, the main difference lies in the fundamental mechanism, i.e., the “dark reactions” can proceed in the absence of catalysts (being just accelerated after catalyst addition), whereas in the case of photocatalysis, usually there is no reaction in the absence of photocatalyst or light.

Regarding the semiconductor photocatalysts, the width of bandgap determines the photoabsorption properties (Klein, 1968; Wilson, 1981; Hamberg et al., 1985; Horan and Blau, 1987; Madhusudan Reddy et al., 2003). Of course, various semiconductors are characterized by different electronic properties, such as bandgap energy and localization of CB and VB. More positive VB and more negative CB mean the higher ability of oxidation and reduction, respectively. Therefore, the wide bandgap results in the efficient redox properties, e.g., simultaneous reduction and oxidation of water. However, the wide bandgap also means the inactivity under visible part of solar spectrum. For example, the most famous semiconductor photocatalyst − titania (titanium(IV) oxide) with wide bandgap of ca. 3.0–3.2 eV (depending on the polymorphic form) is well known from high photocatalytic activity under UV, but also inactivity under visible light (vis). Accordingly, many studies have been performed to modify the structure of titania (and other wide-bandgap semiconductors) via doping, surface modification and coupling with other materials (Khalil et al., 1998; Wu et al., 1998; Zang et al., 2000; Asahi et al., 2001; Ohno et al., 2003; Mitoraj et al., 2007; Zaleska, 2008; Dai et al., 2009; Etacheri et al., 2015; Janczarek et al., 2015; Wang et al., 2016; Babu et al., 2017; Khan et al., 2017; Salehi-Abar and Kazempour, 2017; Sun et al., 2017).

Moreover, in the case of semiconductor photocatalysts, the quantum yields of photocatalytic reactions (even under UV irradiation) do not reach the theoretical value of 100% because of the charge carriers’ recombination (surface or bulk (Herrmann, 1999)). Therefore, numerous studies on the performance improvements have been carried out, focusing on the following aspects: (i) controlled synthesis conditions to improve the properties of photocatalysts (e.g., high crystallinity, a lack of defects, large specific surface area, pure polymorphic forms or fixed ratio of different polymorphs), (ii) morphology architecture, such as the preparation of photocatalysts with exposed facets, different dimensions, advanced morphologies (e.g., inverse opals, nanotubes, nanowires), and (iii) preparation of composite photocatalysts, e.g., by using the metallic or/and nonmetallic elements, and different compounds, to modify the surface or/and the structure of photocatalysts (Bakar and Ribeiro, 2016; Cai et al., 2016; Cheng et al., 2016; Lee et al., 2021; Wang T. M. et al., 2022; Dey et al., 2022; Korosi et al., 2022; Shehab et al., 2022; Shukla and Angappane, 2022). It should be mentioned that for both purposes, i.e., an appearance of vis response and activity enhancement under UV, similar methods have been used for the modifications of wide-bandgap semiconductors, i.e., doping, surface modification, coupling, and nanoarchitecture design (Sun et al., 2019; Huang et al., 2020; Yoshimura et al., 2020; Luo et al., 2021).

**FIGURE 2:**
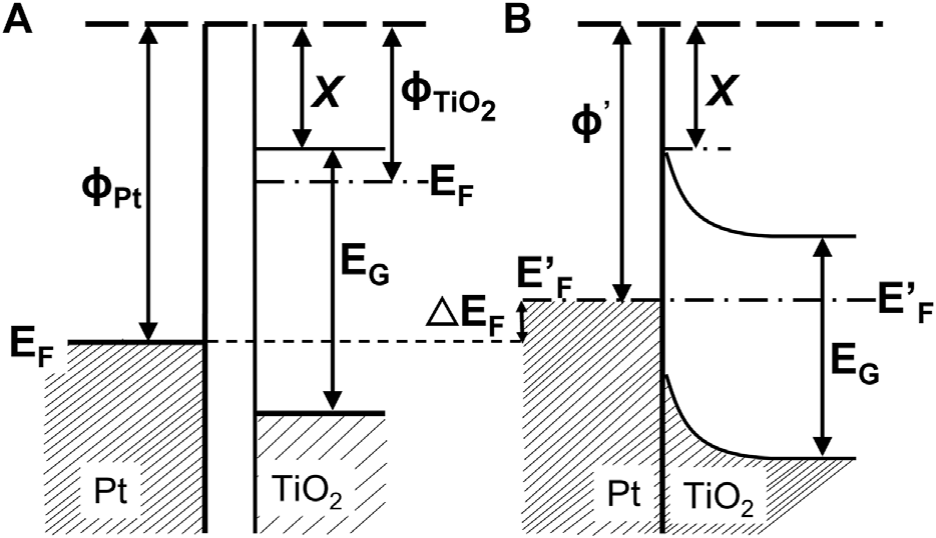
Energy band diagram under UV: before **(A)** and after **(B)** contact between Pt and TiO_2_: X and E_G_ are the electron affinity (ca. 4 eV) and energy bandgap (ca. 3 eV), respectively; Φ_TiO2_ is the work function of illuminated TiO_2_, estimated to be close to the reduced state (ca. 4.6 eV); Φ_Pt_ is the work function of Pt (ca. 5.36 eV); ΔEF = Φ_Pt_ -Φ’_Pt_ is the increase in the Fermi level of Pt after contact with illuminated TiO_2_; drawn based on the report by Disdier et al., 1983.

**FIGURE 3:**
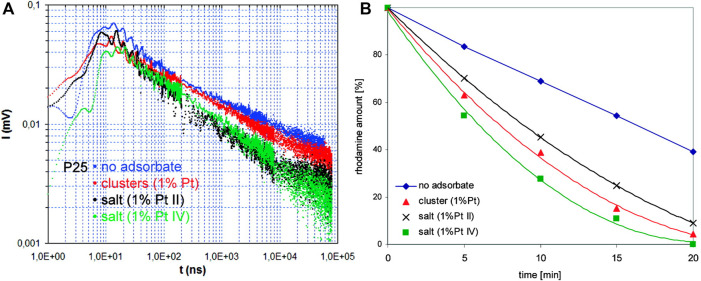
Experimental evidence for electrons’ scavenging by deposited platinum on the titania surface (platinum originated from platinum salts and Chini clusters): **(A)** TRMC results, and **(B)** photocatalytic activity data for oxidative decomposition of rhodamine B. Adapted from (Kowalska et al., 2008) with permission from ACS.

Among various strategies, the surface modification of wide-bandgap semiconductors with noble metals (NMs), such as gold, silver, platinum, has probably been the most popular. Kraeutler and Bard were first who found that platinum could scavenge photogenerated electrons (Figure 1B), and thus hinder the charge carriers’ recombination (Kraeutler and Bard, 1978), as illustrated by Disdier et al., in 1983, and presented here in Figure 2 (Disdier et al., 1983). Since then, huge number of papers have been published on UV-activity enhancement by NMs (Pichat et al., 1981; Pichat et al., 1982; Nishimoto et al., 1985; Jakob et al., 2003; Subramanian et al., 2003; Kowalska et al., 2008; Azri et al., 2014). For example, Hu et al. proved that platinum hinders charge carriers’ recombination by photoluminescence and transient fluorescence spectroscopy, resulting in longer lifetime of photogenerated charge carriers (Hu et al., 2019). Similarly, time-resolved microwave conductivity (TRMC) method was used to show the scavenging of photogenerated electrons by platinum (both originated from inorganic salts and Chini clusters) deposited on the titania surface, which correlates well with the enhanced photocatalytic activity for oxidative decomposition of phenol and rhodamine B (Kowalska et al., 2008), as exemplary presented in Figure 3. Here, improved performance for Pt-modified titania was shown under both UV and vis irradiation. The vis response was explained as originating from the light absorption by Pt-based compounds (sensitization mechanism), i.e., salts, complexes or/and clusters (Macyk et al., 2003; Kisch et al., 2004; Kowalska et al., 2008; Macyk et al., 2010).

**FIGURE 4:**
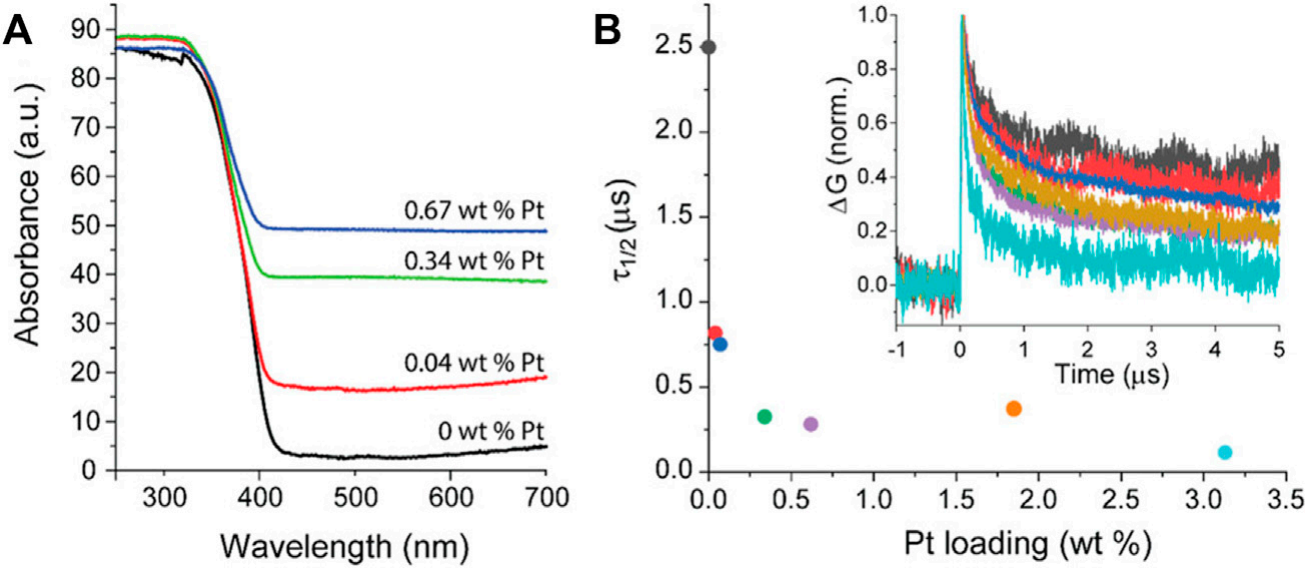
**(A)** DRS spectra for P25 titania loaded with different content of platinum; **(B)** Half time (τ1/2) of the mobile charge carriers measured by TRMC. Inset: transient decay of the mobile charge carrier. Adapted from (Benz et al., 2020), CC-BY-NC-ND license.

In contrast to many reports, Benz et al. have proposed that platinum could work also as a recombination center under anaerobic conditions (Benz et al., 2020). The deposited platinum (0.04–3 wt%) on titania P25 was tested for UV degradation of acid blue nine and rhodamine B in the presence or absence of oxygen in the system. Although, the function of platinum as an electron scavenger has been proven by TRMC method (Figure 4), the decreased activity under anaerobic conditions could suggest that these electrons migrate back to titania. In contrast, under aerobic conditions, superoxide radicals could be efficiently formed on the surface of platinum (electron transfer from titania via platinum to oxygen), as commonly reported. However, it should be mentioned that usually degradation of organic compounds proceeds via oxidative pathways, and thus experiments are performed under aerobic conditions (the participation of reactive oxygen species (ROS); oxygen or air is even continuously bubbled into the reaction system (Gorska et al., 2008; Kowalska et al., 2008)). Additionally, for the reduction pathways, e.g., alcohol dehydrogenation and water splitting, photogenerated electrons are efficiently scavenged by a proton (H^+^). Therefore, obviously, the lack of an electron acceptor (oxygen/proton) should result in charge carriers’ recombination. Moreover, it should be pointed out that platinum could also work as a shield, causing less efficient photon absorption by titania, and thus resulting in lower efficiency, as discussed latter.

In 2005, another property of NMs has been used for the activation of wide-bandgap semiconductors towards vis response, i.e., plasmon resonance, as exemplary shown in Figure 1C. Tian and Tatsuma proposed the electron transfer from photoexcited gold NPs (due to plasmon resonance) to CB of titania, with simultaneous transfer of compensative electrons from the solution to gold NPs (Tian and Tatsuma, 2005). Since then, many reports showed vis activity of NM modified wide-bandgap semiconductors, resulting from plasmonic activation, known as “plasmonic photocatalysis” (Furube et al., 2007; Kowalska et al., 2009; Mukherjee et al., 2013; Panayotov et al., 2013; Sugawa et al., 2015; Verbruggen, 2015; Verma et al., 2018; Endo-Kimura and Kowalska, 2020; Raja-Mogan et al., 2020; Wei et al., 2020).

Moreover, another function of NMs should also be pointed out, i.e., co-catalytic. In the case of some reactions, pristine photocatalysts might be almost inactive, e.g., alcohol dehydrogenation and water splitting. Titania and other wide-bandgap semiconductors are hardly active due to high overvoltage for both hydrogen and oxygen evolution (Bandara et al., 2005; Abe et al., 2008; Kowalska et al., 2015; Takanabe, 2017; Yoshiiri et al., 2022). Accordingly, it has been found that NM co-catalysts, deposited on the surface of wide-bandgap semiconductors, are highly efficient for hydrogen evolution reactions. For example, Pt-deposition on P25 titania (commercial sample with one of the highest photocatalytic activities) results in activity enhancement by more than one order in magnitude (Wang et al., 2018; Wang K. et al., 2022; Paszkiewicz et al., 2022). Obviously, the properties of these deposits as well as the interface between NMs and semiconductor are decisive for the overall performance. However, the contrary results might be found in the literature. Additionally, the oxidation state of platinum could also play important impact on the photocatalytic activity. Accordingly, this review aims to revised various studies on the most active NM-modified semiconductors, i.e., platinum-modified titania, to clarify the key factors of photocatalytic activity, and to propose what properties are the most recommended for specific reactions.

Considering platinum-modified titania samples, more than 5000 papers could be found in Web of Science (2022/06/16 search for: (i) Pt/TiO_2_, (ii) platinum and titania, and (iii) platinum and titanium dioxide). Most of the studies discuss titania samples modified with nanoparticles (NPs) of platinum for various photocatalytic reactions. However, many reports deal with “dark” activity of platinum (not photocatalytic) where titania is only the support − inert or participating slightly in the overall mechanism, e.g., Pt/TiO_2_ has been used as catalyst, electrocatalyst, sonocatalyst, sensor and for resistive switching memory (RSM) devices (Makouangou et al., 1994; Fukuoka et al., 1999; Lee et al., 2009; Song et al., 2011; Takakusagi et al., 2014; Dhanasekaran et al., 2016; Komanoya et al., 2016; Touchy et al., 2016; Ammal and Heyden, 2017; Kon et al., 2017; Nakajima et al., 2021). Additionally, there are some reports suggesting the doping of titania with platinum (Ambrozova et al., 2018; Zener et al., 2018; Ihnatiuk et al., 2020), and photocatalytic activity of titania modified with platinum single atoms (Qin et al., 2022). Moreover, the surface modification of titania with other forms of platinum (not NPs), such as compounds, complexes and clusters, have also been proposed for both UV and vis activity (Macyk and Kisch, 2001; Kisch et al., 2004; Mitoraj et al., 2007; Kowalska et al., 2008; Kisch, 2011; Khnayzer et al., 2012). However, because of huge number of various papers on Pt/TiO_2_, and thus impossibility to comprehensively present them in one review paper, only titania photocatalysts modified with platinum NPs are discussed in detail in this review.

## Synthesis of platinum-modified titania photocatalysts

The large number of scientific papers on Pt/TiO_2_ photocatalysts corresponds obviously to various synthesis procedures of their preparation. Considering the titania aspect, both commercial and self-synthesized samples are used. Here, the most typical methods/samples are titania P25 (from Evonik/Degussa; composed of anatase (78%), rutile (14%) and non-crystalline phase (8%); with specific surface area of ca. 52 m^2^ g^−1^, and one of the highest photocatalytic activities in various reaction systems (Ohtani et al., 2010; Wang et al., 2018)) and hydrolysis of titania precursor (titanium alkoxides: isopropoxide and butoxide), respectively (Hufschmidt et al., 2002; Zhao et al., 2002; Coleman et al., 2005; Kowalska et al., 2008; Rosario and Pereira, 2014; Zielinska-Jurek et al., 2015; Haselmann and Eder, 2017; Yurdakal et al., 2017). Of course, other synthesis methods were also proposed, such as, gas-phase reaction (e.g., titanium(IV) chloride with oxygen), hydrothermal reaction (e.g., from titanate nanowires), and anodization of titanium (Nam and Han, 2007; Khan et al., 2008; Amano et al., 2010; Wei et al., 2014; Janczarek et al., 2016; Zielinska-Jurek et al., 2019; Momeni and Jalili, 2022). Then, either pre-synthesized Pt colloid/NPs or its precursor (usually chloroplatinic acid hexahydrate − H_2_PtCl_6_ · 6H_2_O) was used for the modification of the titania surface by various methods, including photodeposition (the most popular), impregnation, microemulsion, chemical and thermal reduction, electrostatic adsorption, ion exchange, incipient wetness, atomic layer deposition (ALD), complete decomposition of surface-anchored platinum complexes (Pt (dcbpy)Cl_2_; dcbpy = 4,4′-dicarboxy-2,2′-bipyridine), and sputtering (Herrmann et al., 1986; Li et al., 2006; Nam and Han, 2007; Li et al., 2013; Bear et al., 2015; Samad et al., 2015; Shahgaldi and Hamelin, 2015; Zielinska-Jurek et al., 2015; Zielinska-Jurek et al., 2019; Benz et al., 2020). In some cases, titania was pre-treated by heating, sonication or irradiation. Heating was used to: (i) change the properties, e.g., crystallinity, crystal composition, specific surface area, (ii) stabilize the titania surface, and (iii) allow better adsorption of platinum (Li et al., 2006; Bear et al., 2015). Whereas, ultrasonication and UV irradiation were proposed for the generation of oxygen vacancies in the bulk and on the surface, respectively (Haselmann and Eder, 2017). It has also been shown that the kind of platinum salt might influence the resultant properties. For example, chloroplatinate ions in the solution reach the reduction sites via diffusion (photodeposition method), causing Pt enlargement of islets, whereas platinum compounds that strongly adsorbs onto titania (e.g., PtI_2_^−6^) cause that [Pt] in solution phase is minimal (Senevirathna et al., 2006). In contrast, different method of deposition even for the same platinum salt (chloroplatinate acid) might result in either uniform distribution of fine Pt NPs or their aggregation, as shown for impregnation ([PtCl_6_]^2−^ adsorbs on the positively charged titania surface quite uniformly due to the high acidity of the solution.) and photodeposition, respectively (Kozlova et al., 2011). Additionally, some reports suggest also the post-treatment operations (annealing, sonication, H_2_ reduction) for: (i) stabilization and/or better connection between platinum and titania, (ii) increasing specific surface area, (iii) strong metal-support interaction (SMSI) effect (e.g., electron migration from titania to platinum), (iv) uniform distribution, and (v) the change of the oxidation state of platinum, e.g., from zero-valent to divalent, resulting from the diffusion of platinum from its NPs into the lattice of titania grains and substitution for Ti^4+^ (Colmenares et al., 2006; Li et al., 2006; Khan et al., 2008; Colmenares et al., 2011; Dessal et al., 2019).

Photodeposition of platinum on the titania surface is probably the most common (examples shown in Table 1) due to many advantages, such as:

- short time (for platinum ca. 5–10 min of induction time − complete deposition (Wei et al., 2017a)),
- a good contact between titania and platinum (Since metal cations are reduced by photogenerated electrons directly on the titania surface.),
- complete deposition of all platinum cations (from platinum salt),
- low cost and simple procedure (Only irradiation source is necessary—daylight could also be applied.),
- the ability to monitor *in-situ* the photocatalytic activity during platinum deposition, e.g., by hydrogen evolution (Senevirathna et al., 2006).

**TABLE 1:** The examples of Pt/TiO_2_ photocatalysts’ preparation by photodeposition method.

Platinum			Titania				Photodeposition Condition	References
Content (%)	Size (nm)	Distribu-tion	BET (m^2^/g)	Size (nm)		Type		
	PPA			PPA	SPA			
0.2	nd	nd	71–131	2–10	nd	A, AR, (pH depending) homemade	UV, 7 h	Huang et al. (2018)
0–0.3	nd	nd	nd	nd	nd	A/R, P25	UV, anaerobic, 1 h	Senevirathna et al. (2006)
0.50	4	nd	49	20	nd	A/R, P25	UV, 12 h	Cubillos-Lobo et al. (2017)
1	4	nd	nd	nd	10	A, homemade RGO-TiO2	UV, 3 h	Shinde et al. (2018)
0.2–3.0	<2	nd	nd	nd	nd	A, homemade	UV, 8 h	Zheng et al. (2009a)
2.1	2–3	nd	17.9	45 for homemade	nd	A/R P25, A Hombikat UV100, A homemade	UV, anaerobic, 6 h	López et al. (2015)
0.08–1.8	nd	nd	54	ND	nd	A/R, P25	solar simulator	Chowdhury et al. (2013)
0.5–2.0	3–12	nd	64	14.8	nd	A, homemade	UV, 3 h	Selvam and Swaminathan, (2011)
0.50	nd	nd	ca.70	ND	nd	A, homemade	UV, 2 h	Vaiano et al. (2018)
0.5 and 2	3–5, 10	agg	ca.50	ND	nd	A, homemade	UV, 15–240 min	Murcia et al. (2014)
0.5 and 1	1.5	uniform	300	8	nd	A, Hombikat UV100	UV	Al-Madanat et al. (2021)
0.1–1.0	0.5–0.7	agg	**—**	**—**	**—**	A/R, modified P25	455 nm, 70 min	Vasilchenko et al. (2020)
	—	—	56	5–10	nd	A, zeolite-TiO2 (P25)	365 nm, 2 h	López-Tenllado et al. (2022)
0.9	1–5	Nd	nd	nd	nd	P25	UV, 0.5–1 h	Bamwenda et al. (1997)

A—anatase, agg.—aggregated, nd-not determined, R-rutile, RGO—reduced graphene oxide.

Photodeposition method is based on the main feature of photocatalysis, that is the formation of charge carriers under irradiation, i.e., the photogenerated electrons reduce platinum cations. Usually, photodeposition is performed in the absence of oxygen (to avoid electron scavenging) and in the presence of a hole scavenger, here, methanol is the most popular (to limit hole-electron recombination). After the reaction completion, Pt/TiO_2_ is washed (usually first with methanol to remove all adsorbed organic products from the hole scavenger oxidation, and then with water), dried (Common drying in the presence of air is used as both components are not easily oxidized, in the contrast to silver- or copper-modified samples when freeze drying is recommended (Janczarek et al., 2017)), and ground. Since in the case of platinum, the photodeposition is very fast (completed within few minutes), which might result in platinum aggregation, the modified photodeposition method has also been proposed. For example, photodeposition performed in the initial presence of oxygen or air in the system (aerobic system) might be used, i.e., the samples containing titania, platinum salt and methanol are sealed but the pre-bubbling with inert gas (e.g., argon or nitrogen) is not applied. Accordingly, during irradiation the photogenerated electrons are simultaneously scavenged by oxygen and also used to reduce platinum cations, which causes the formation of smaller and more uniformly distributed platinum NPs (Wei et al., 2017a; Paszkiewicz et al., 2022). However, it might also mean the change in the surface oxidation state of platinum. Indeed, samples prepared in the initial presence of oxygen contain larger content of positively charged platinum than those prepared under anaerobic conditions (Wei et al., 2017a; Paszkiewicz et al., 2022).

The method of synthesis obviously governs the resultant properties of Pt/TiO_2_ photocatalysts. For example, smaller and more uniformly distributed Pt NPs have been deposited on the titania by: (i) decomposition of surface-anchored platinum complexes than photodeposition method (Li et al., 2013), (ii) ion exchange (2 nm) than impregnation (12 nm) method (Khan et al., 2008), (iii) photodeposition in the initial presence of oxygen than under anaerobic conditions (Wei et al., 2017a; Paszkiewicz et al., 2022), (iv) thermal reduction (2, 5 and 9 nm) than chemical reduction (3, 6 and 17 nm, respectively, on different titania samples: ST01-fine anatase, P25 and decahedral anatase particles (DAP)) (Zielinska-Jurek et al., 2019).

Similarly, the procedure of platinum deposition obviously influences the surface oxidation state of platinum. For example, it has been shown that chemical reduction and photodeposition methods result in the preparation of samples with mainly zero-valent and positively charged platinum NPs, respectively (Kozlova et al., 2011). However, other reports show that photodeposition causes the formation of mainly zero-valent platinum (Wei et al., 2018). Therefore, it is though that the slight change in the photodeposition conditions (irradiation source, light intensity and the absence/presence of oxygen) might result in significant change in the properties, and thus in the resultant activities. Other studies indicate that more positively charged platinum is obtained when photodeposition is performed in the initial presence of oxygen in the system than that performed under anaerobic conditions (Wei et al., 2017a; Paszkiewicz et al., 2022). Moreover, post-treatment operations, such as thermal treatment, might cause the change of oxidation state of platinum, e.g., from Pt^0^ to Pt^2+^, due to titania doping with diffused platinum from its NPs (Li et al., 2006).

## Property-governed activity of platinum-modified titania photocatalysts

Like pristine titania, platinum-modified titania photocatalysts have been applied for plenty different reactions, including environmental purification (water/air purification, wastewater treatment, self-cleaning surfaces), energy conversion (water splitting, photocurrent generation), and organic synthesis, as shown in Table 2. For example, Avila et al. found in 1998 that Pt/TiO_2_ was efficient for the destruction of traces of organic pollutants present in gaseous emissions (Avila et al., 1998). Then, various studies have been performed under UV irradiation. However, considering the high cost of artificial sources of irradiation, the recent study has mainly focused on the activity tests performed under natural solar light (or solar simulator) and the sole vis irradiation. Indeed, many reports have already proven that titania modification with platinum results in vis response (Kowalska et al., 2008; Zielinska-Jurek et al., 2015; Zielinska-Jurek et al., 2019). Moreover, considering high costs of platinum, the experiments with low its content (below 1 wt%) have been prioritized. In most cases, the application of platinum has significantly improved the photocatalytic activity of titania. For example, the reaction rate of toluene conversion has increased by ca. four times after platinum deposition on titania (Avila et al., 1998). The most significant activity enhancement is obviously observed for gas evolution reactions and activity under vis since pristine titania is practically inactive (as discussed in Introduction).

**TABLE 2:** The examples of enhanced photocatalytic activity after platinum deposition on titania.

Pt/TiO_2_		Tested reaction		Main findings	References
Content (%)	Type	UV	Vis		
0.20	A, A/R	dye decolorization	—	low charge recombination rate	Huang et al. (2018)
0–0.3	A/R	CH_3_OH dehydrog	—	increased quantum efficiency from 12.5 to 42.5%	Senevirathna et al. (2006)
0.50	A/R	phenol and methyl orange degr	phenol and methyl orange photodegradation	Pt on sulphated TiO_2_ - vis response	Cubillos-Lobo et al. (2017)
1	A	CH_3_OH dehydrog., propranolol degr	—	94% under solar	Shinde et al. (2018)
0.2–3.0	A	H_2_ generation from HAc	—	optimized Pt amount	Zheng et al. (2009a)
2.1	A	H_2_ generation	—	methanol > ethanol > ethyleneglycol > glycerol	López et al. (2015)
0.08–1.8	A/R	H_2_ generation from formaldehyde (40–4600 ppm)	H_2_ generation from formaldehyde (40–4600 ppm)	Langmuir-type model; platinum (wt%), catalyst dose, light intensity, and initial concentration	Chowdhury et al. (2013)
0.5–2.0	A	synthesis of 2-methylquinolines	—	improved activity	Selvam and Swaminathan, (2011)
0.5	A	H_2_ generation from glycerol	—	improved activity	Vaiano et al. (2018)
0.5 and 2	A	phenol degr	—	small Pt NPs - phenolates formation; large Pt NPs - surface interaction	Murcia et al. (2014)
0.5 and 1.0	A	photocatalytic reforming of naphthalene and methanol	—	strong interaction between Pt NPs and TiO_2_ surface	Al-Madanat et al. (2021)
0.1–1.9	A/R	CH_3_OH dehydrog	—	the low Pt content—high activity	Vasilchenko et al. (2020)
	A	glycerol degr	—	increased activity	Lopez-Tenllado et al. (2022)
1	A	—	HCHO degr	increased activity	Zhu and Wu, (2015)
1%	A/R	hydrogenation of phenylacetylene to styrene		high selectivity and conversion	Lian et al. (2020)

A—anatase, degr—degradation, dehydrog.—dehydrogenation, R-rutile.

The most important question is: What parameters and properties should be considered to design efficient photocatalysts, based on platinum-modified titania? Of course, methods of preparation are crucial since they govern the resultant properties. Considering the properties, it seems that three are the most important: the platinum content, the platinum size (and its distribution) and its surface oxidation state. All these aspects are shortly presented and discussed below.

In the case of platinum content, the contrary reports have been published, suggesting that smaller or larger amount is better (Hufschmidt et al., 2002). However, usually the optimal amount (Table 3) has been suggested, ranging from 0.06 to 2 wt%, e.g., (i) 1.5 wt% for methyl orange degradation under UV/vis (Hu et al., 2012); (ii) 0.025 wt% for degradation of dichloromethane under UV (Ma et al., 2011), (iii) 0.5 wt% for UV methanol dehydrogenation (Ahmed et al., 2014), (iv) 0.2 wt%, 1 wt% or 2 wt% for methanol dehydrogenation, depending on the titania type (Wang et al., 2018), (v) 0.1 wt% for phenol degradation under UV and vis irradiation (Zielinska-Jurek et al., 2019), (vi) 0.057 wt%, 0.3 wt% and 2 wt% for methanol dehydrogenation, depending on the method of Pt deposition (Senevirathna et al., 2006), (viii) 0.1 wt% for hydrogen generation from acetic acid under UV (Zheng et al., 2009), (ix) 0.5 wt% for hydrogen evolution under simulated solar radiation (Hu et al., 2019), (x) 1 wt% for selective hydrogenation of phenylacetylene to styrene under UV (Lian et al., 2020), and (xi) 0.2 wt% for UV synthesis of benzimidazoles (Shiraishi et al., 2010). Interestingly, it has been found that the reaction conditions (pH value), irradiation intensity, the type of titania, kind of tested compounds and their concentration are also decisive, influencing the optimal content of platinum (Hufschmidt et al., 2002; Ma et al., 2011). The type of titania relates to the surface properties, such as specific surface area and crystallinity, and thus obviously different number of platinum NPs should be optimal for titania with different properties.

**TABLE 3:** Exemplary results for the optimization of platinum content.

Tested Content of Pt	Optimal Pt amount (wt%)	Tested Compound	Irradiation	References
0.2–1.0 wt%	1.0	Acetic acid	UV/vis	Zheng et al. (2009a)
0–1.8 wt%	0.25	Formaldehyde	simulated solar and vis	Chowdhury et al. (2013)
0.005–2 wt%	0.2, 1.0, 2.0	methanol	UV/vis	Wang et al. (2018)
2–30 mg L^−1^	0.057, 0.3 and 2.0	methanol	UV	Senevirathna et al. (2006)
0.5%, 2.0 wt%	0.5	phenol/methyl orange	UV	Murcia et al. (2014)

**FIGURE 5:**
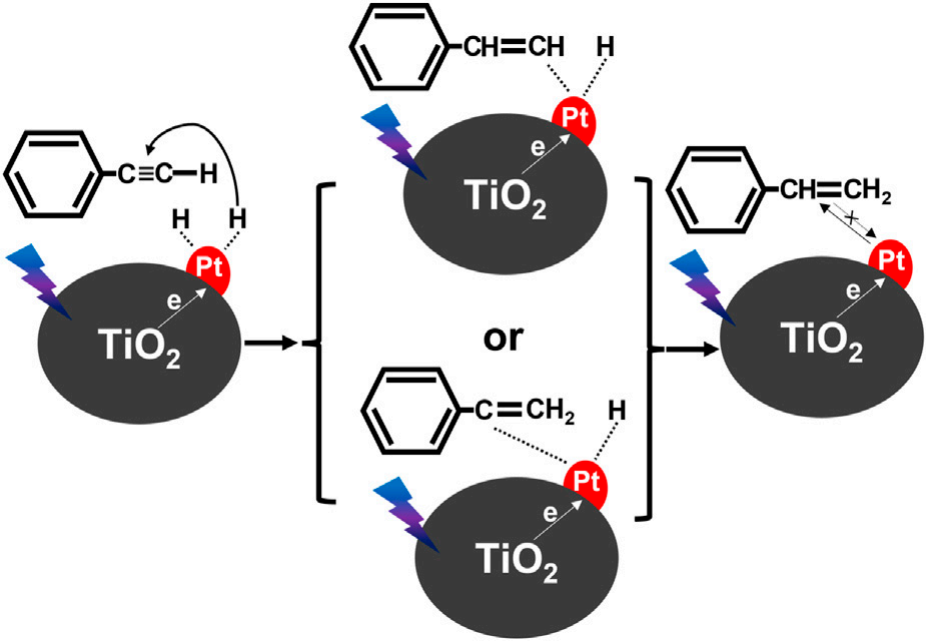
Proposed photocatalytic reaction mechanism for semi-hydrogenation of PLE over Pt/TiO_2_; drawn based on the report by (Lian et al., 2020).

In the case of organic synthesis, it has been proposed that the amount of deposited platinum is a decisive factor for both high conversion and selectivity. For example, Lian et al. found that 1 wt% of Pt deposited on titania was the most efficient for the photocatalytic conversion of phenylacetylene (PLE) to styrene (STE) under monochromatic light (385 nm) irradiation (Lian et al., 2020), reaching 92.4% (conversion) and 91.3% (selectivity). It has been proposed that photogenerated electrons (from titania) migrate to Pt NPs, whereas methanol as both hydrogenation source and electron donor (holes’ scavenger) dissociates (H^+^). Then, active hydrogen species, formed on the surface of platinum, hydrogenate PLE (H-Pt), and thus formed STE detaches from the photocatalyst surface, as shown in Figure 5. It has been proposed that the selectivity towards STE is caused by the increased electron density of photocatalyst (electron cumulation on platinum), decreasing the adsorption strength of intermediate STE.

It should be pointed out that usually only several different photocatalysts have been tested, and thus with only two to four different contents of platinum. Therefore, it is hardly possible to classify these studies as “optimization”. Fortunately, there are also comprehensive reports showing the influence of platinum content in the wide range of concentrations. For example, Zheng et al. investigated nine different contents of platinum (0.2, 0.4, 0.6, 0.8, 1.0, 1.2, 1.4, 2.0 and 3.0 wt%) deposited on self-synthesized titania, with 1.0 wt% being the most active for hydrogen evolution from acetic acid solution (Zheng et al., 2009). Similarly, nine different concentrations of platinum salt have been investigated by Senevirathna et al. to find that 4.5 mg L^−1^ is the most recommended for methanol dehydrogenation under UV (Senevirathna et al., 2006). Table 3 presents exemplary results for the optimization of platinum content on the titania surface.

**FIGURE 6:**
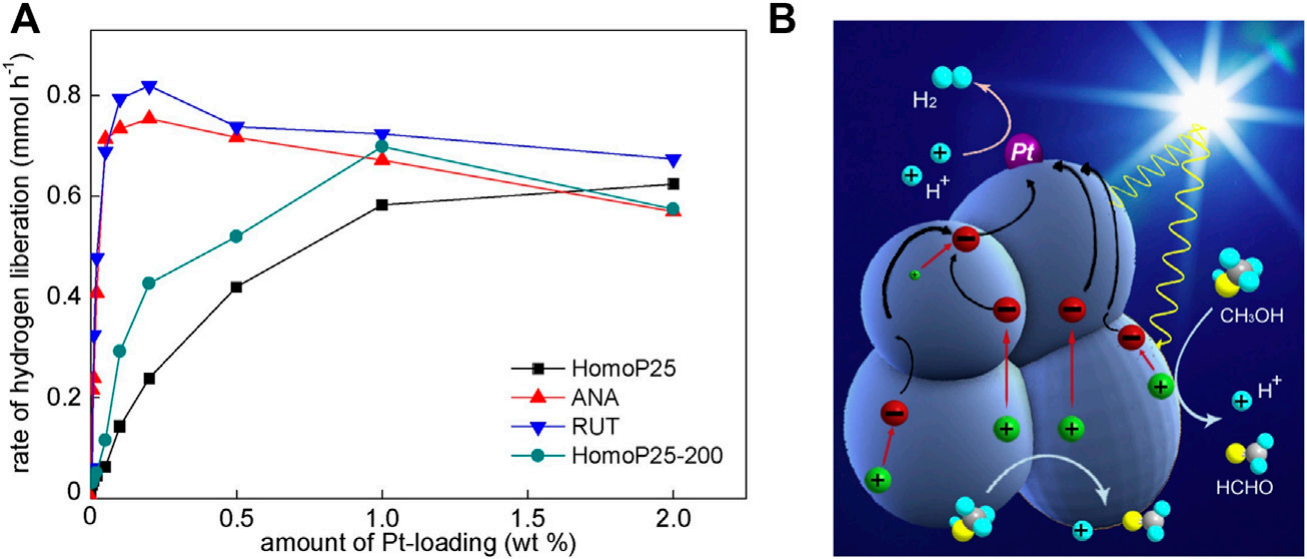
**(A)** The influence of platinum content on the photocatalytic activity during methanol dehydrogenation; **(B)** the schematic image showing the IPET in aggregated single-phase titania particles. Adapted from (Wang et al., 2018) with permission from Elsevier.

In respect of this, it is thought that our previous report on platinum-modified titania, in which 36 samples have been tested, might be also very meaningful (Wang et al., 2018). In this study, nine different amounts of platinum (0.05, 0.01, 0.02, 0.05, 0.1, 0.2, 0.5, 1.0 and 2.0 wt%) and four titania samples, originated from the famous P25 titania, i.e., homogenized P25 (HomoP25), homogenized P25 thermally treated at 200 °C (HomoP25-200), anatase isolated from HomoP25 and purified by annealing at 200 °C (ANA) and rutile isolated from HomoP25 and purified by NaOH washing and annealing at 200 °C (RUT), were tested. It has been found that optimal content of platinum (tested during methanol dehydrogenation) depends on the titania feature, reaching 0.2 wt% for ANA and RUT, 1 wt% for HomoP25-200 and 2 wt% for HomoP25, as shown in Figure 6A. It should be pointed out that here, ANA and RUT have been obtained from HomoP25, and thus these samples are characterized by similar properties that those in P25. It has been concluded that much different activities and optimal properties come from the aggregation of titania, caused by thermal treatment (used for samples’ purification). It should be remembered that in the case of methanol dehydrogenation one platinum deposit on one titania particle (e.g., aggregate) is sufficient for efficient hydrogen evolution (Ohtani et al., 1997), whereas an increase in platinum NPs’ number might cause the “shielding effect”, i.e., the competition between titania and platinum for photons (also known as “light-shading effect” (Lian et al., 2020)). Accordingly, it has been concluded that interparticle electron transfer (IPET; photogenerated electrons within one aggregate moving to one platinum deposit) is also possible within the same polymorph (anatase-anatase, rutile-rutile), and it has been named as Homo-IPET, as illustrated in Figure 6B.

Interesting study has been presented by Senevirathna et al. for P25 titania modified with platinum by three methods: photodeposition from hexaiodoplatinic acid, photodeposition from chloroplatinic acid and impregnation (chloroplatinic acid) with thermal treatment (Senevirathna et al., 2006). It has been shown that optimal content of platinum depends on both the method of preparation and the platinum salt, reaching 0.057 wt%, 0.3 wt% and 2 wt%, respectively. It has been proposed that the uniform distribution of fine Pt NPs, allowed by strong adsorption of PtI_2_^−6^ on the titania surface, results in the highest photocatalytic activity.

**FIGURE 7:**
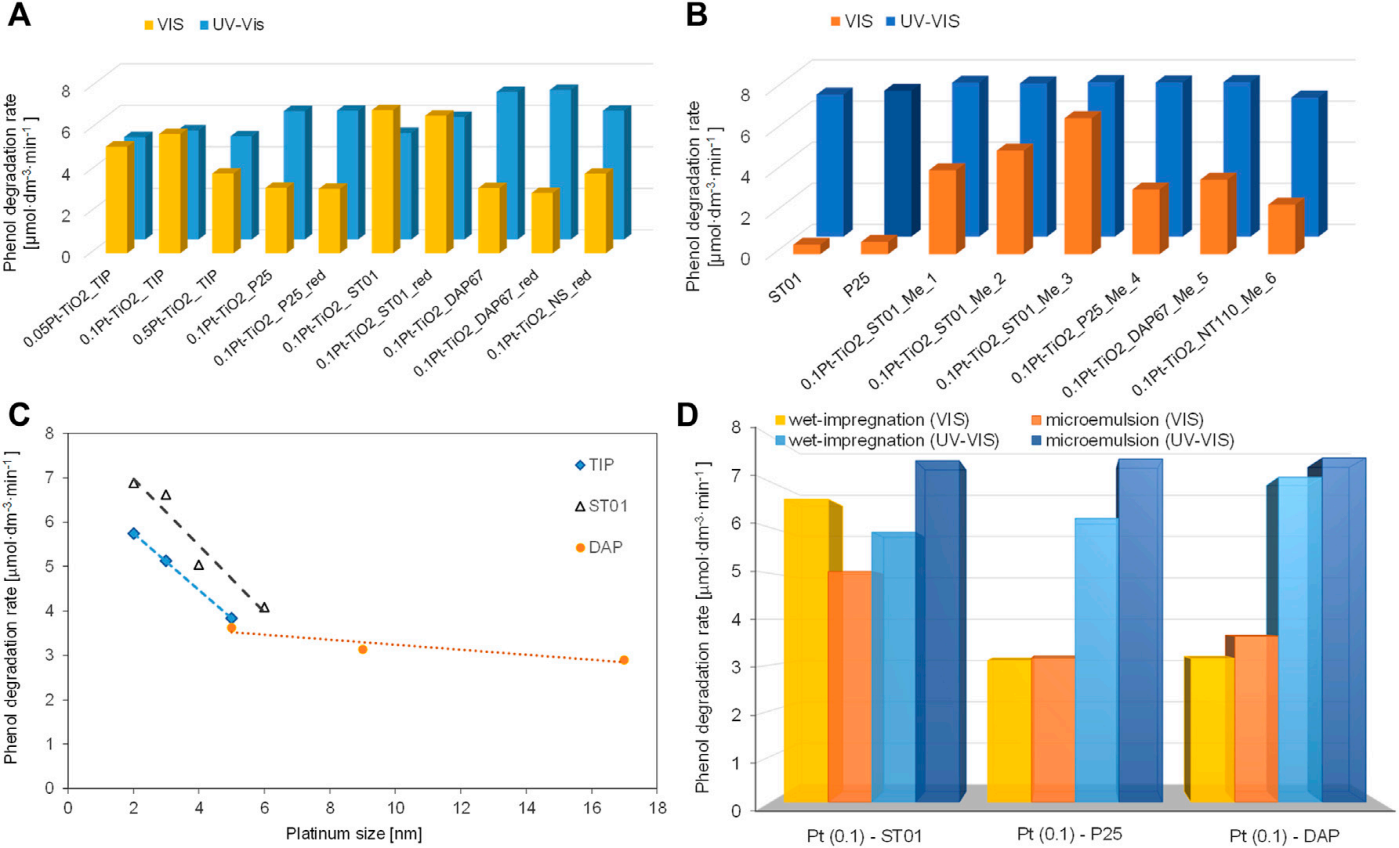
**(A,B)** Photocatalytic activity of Pt-TiO_2_ samples obtained by: **(A)** wet-impregnation method, **(B)** microemulsion method; **(C)** Correlation between Pt NPs’ size and photocatalytic activity of TIP (self-synthesized titania from titanium isopropoxide), ST01 (commercial samples of fine anatase) and DAP, **(D)** Comparison of UV-vis and visible-light photocatalytic activity for Pt-modified titania samples. Adapted from (Zielinska-Jurek et al., 2019), under Creative Common CC BY license.

Similar to platinum content, the contrary reports on the best size of platinum deposits have been reported, i.e., showing that smaller, larger or optimal size is the most recommended. It should be pointed out that the opposite results could be caused by different application, i.e., photocatalytic reactions under UV or vis irradiation, where better distribution (fine NPs uniformly deposited on titania) or aggregates of more efficient light harvesting ability, respectively, are recommended (Villani et al., 2006; Ting et al., 2015; Wei et al., 2017a; Wei et al., 2017b; Zielinska-Jurek et al., 2019). For example, Li et al. have shown that smaller and highly dispersed platinum NPs on titania result in higher activity for hydrogen evolution both under UV and vis irradiation (vis activity due to co-adsorbed ruthenium complex) (Li et al., 2013). Zielinska-Jurek et al. investigated simultaneously the influence of various factors on the resultant activity, i.e., the properties of titania (morphology, specific surface area, crystal size, etc.), the preparation method (commercial and self-synthesized titania by different methods for both titania synthesis and platinum deposition: hydrolysis, gas-phase, micoremulsion, impregnation, thermal and chemical reduction), platinum properties (size and shape: spherical or cubic) and the contact between titania and platinum (Zielinska-Jurek et al., 2019). In contrast to other reports, suggesting that under vis irradiation the larger and polydisperse NM particles are important for efficient light harvesting, i.e., broad plasmon peak (Kowalska et al., 2009; Kowalska et al., 2010; Wei et al., 2017a; Wei et al., 2017b), higher activity with a decrase in Pt size was shown, as presented in Figure 7. However, it should be remembered that titania properties (and even its type) have also been changed, and thus the least active sample under vis is characterized by largest Pt NPs deposited on faceted anatase particles with decahedral shape (DAP). In another study, it has been proven that this morphology, despite being highly active under UV (one of the most active titania photocatalysts (Amano et al., 2009; Tachikawa et al., 2011; Janczarek et al., 2016)), is detrimental for vis activity of plasmonic photocatalysts, because of the fast back “hot” electron transfer (Au→TiO_2_→Au) (Wei et al., 2019). Therefore, it is thought that for fruitful discussion it is recommended to change only one factor, e.g., platinum size/amount or titania type/properties, and thus direct correlation between only one specific property and activity could be drawn.

Interestingly, it has also been proposed that smaller Pt NPs (ca. 2 nm) on titania nanotubes obtained by ion exchange method than those prepared by impregnation (ca. 12 nm) result in bandgap narrowing, and thus vis response—stoichiometric generation of hydrogen and oxygen (water splitting) (Khan et al., 2008).

Next, the oxidation state of platinum should also be discussed. Of course, the platinum NP means that it is composed of zero-valent platinum, but at the same the charge on its surface could vary. Here, similarly to the influence of platinum content and Pt NPs’ size, the contrary reports could be found, suggesting that zero-valent or positively charged platinum is better. For example, Li et al. have shown that the formation of positively charged platinum (e.g., by the thermally initiated diffusion of platinum from its NPs inside the titania lattice) results in higher photocatalytic activity for CO oxidation due to a decrease in the contacted resistance on the interface, being beneficial for the transfer of the photo-generated electron (Li et al., 2006). Similarly, positively charged platinum NPs (Pt^2+^ and Pt^4+^) deposited on P25 titania exhibit higher antimicrobial activity against Gram-negative bacteria in rotating magnetic field (instead of UV irradiation), possibly due to electrostatic attractions (Paszkiewicz et al., 2022). In contrast, Gram-positive bacteria are more sensitive to the photocatalyst with larger content of zero-valent platinum. Furthermore, in the case of gas-phase UV photocatalytic oxidation of dimethyl methylphosphonate (Kozlova et al., 2011) and methanol dehydrogenation (Wei et al., 2017a), samples with the larger content of zero-valent platinum show to be more efficient. However, in the case of oxidative decomposition of acetic acid, the samples with larger content of positively charged platinum show higher photocatalytic activity under UV (Wei et al., 2017a; Paszkiewicz et al., 2022). Similarly, in the case of organic synthesis, the selectivity towards organic products, e.g., during conversion of phenylacetylene to styrene, is preferable when oxidized form of platinum is applied (Lian et al., 2020).

**FIGURE 8:**
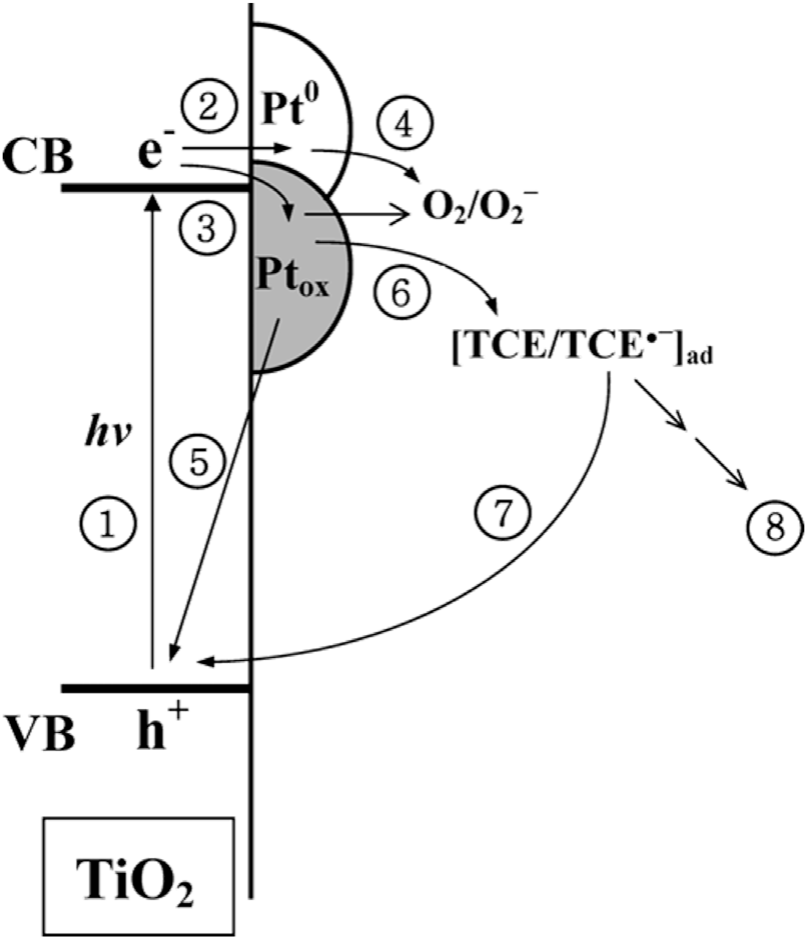
The schematic drawing showing the proposed photoinduced electron transfer paths on platinum-modified titania (Pt_ox_-oxidized form of platinum; Pt^0^—zero-valent platinum) in the presence of TCE; The numbers indicate major electronic pathways: (1) band gap excitation; (2) electron trapping in the Pt^0^ phase; (3) electron trapping in the Pt_ox_ phase; (4) trapped electron transfer to O_2_; (5) Pt_ox_-mediated recombination; (6) trapped electron transfer to TCE; (7) TCE-mediated recombination; (8) reductively initiated degradation of TCE. Adapted with permission from (Lee and Choi, 2005) with permission from ACS.

Very interesting study was performed by Lee and Choi on different Pt/TiO_2_ photocatalysts for photocatalytic degradation of chlorinated organic compounds (trichloroethylene (TCE), perchloroethylene (PCE), dichloroacetate (DC)) under UV irradiation (Lee and Choi, 2005). They found that among various properties, the oxidation state of platinum was the most decisive, and thus samples with larger content of zero-valent platinum were the most active for all tested compounds. Positively charged platinum (Pt_Ox_) strongly inhibited the oxidation of TCE and PCE, but it was still more reactive than pristine titania for PCD. It was proposed, based on the photoelectrochemical studies (lower photocurrents for an electrode with positively charged platinum), that Pt_Ox_ species worked as a recombination center. The mediated charge recombination on Pt_Ox_ through the redox cycle of TCE was proposed, as shown in Figure 8. Accordingly, it has been concluded that the effect of platinum in photocatalysis is highly substrate specific, and both the properties of platinum and the interactions between platinum and substrate influence the overall activity.

## Conclusion

Many attempts have been performed to find the property-governed activity for Pt/TiO_2_ photocatalysts. In those studies, different factors have been considered, such as platinum content, platinum properties (size, aggregation/uniform distribution, oxidation state), titania properties (size, crystallinity, specific surface area, polymorphic form, defect density, morphology, surface characteristics) and the contact between platinum and titania. Unfortunately, usually only few factors have been varied at the same time. The most common studies have been performed for only several different samples, e.g., (i) pristine titania and titania modified with two/three different contents of platinum, (ii) two-four different titania samples modified with the same content of platinum, (iii) pristine titania and titania modified with platinum by two/three different methods. Additionally, though the properties of Pt/TiO_2_ samples have been well characterized in many cases by various advanced methods, usually these properties have neither been controlled nor designed. Moreover, the photocatalytic activities have been tested for quite different reactions and in different systems (photoreactors, light intensity, spectrum range). It should be pointed out that even studies showing quantum yields are difficult for comparison when performed in different laboratories since only apparent quantum yields are usually estimated (emitted but not absorbed photons). Accordingly, even after so many years and huge number of published reports in the field, it is still hardly possible to draw the general conclusions on the property-governed activity of platinum-modified titania.

However, it might be proposed that in many cases, fine platinum NPs uniformly distributed on the titania support results in high photocatalytic activity. Moreover, the preparation of this kind of photocatalyst might cause that optimal content of platinum is really low (below 1 wt%), and thus the photocatalyst even containing platinum could be reasonably cheap. Additionally, the aggregation of titania particles might also be recommended to decrease the necessary amount of platinum (e.g., 0.2 wt%), since IPET between same titania NPs (in one aggregate) allows an efficient electron transfer to platinum deposits. In the case of oxidation state of platinum, it is though that very important is the reagent, and thus its easy adsorption on the photocatalyst surface. Therefore, either positively charged or zero-valent platinum could be beneficial for the specific reaction.

Finally, it might be concluded that platinum is probably the most efficient modifier of titania, resulting in significant enhancement of quantum yields of photocatalytic reactions. Additionally, platinum-modified titania has also shown activity under visible range of solar spectrum, and thus being highly efficient under natural solar radiation. However, the price of platinum and its possible negative influence on the environment (e.g., in the case of the leakage from the photocatalyst surface) are the main shortcomings of its application. Accordingly, a decrease in its content and strong adsorption to titania must be achieved for possible commercial application. Additionally, there is a lack of comprehensive studies, in which only one property could be controlled and analyzed in detail for various photocatalytic reactions. Accordingly, our research is focused on this aspect now, and it is thought that the final conclusions on the property-governed activity of platinum-modified titania could be drawn in the nearest future.
